# Elevated NT-Pro-Brain Natriuretic Peptide Level Is Independently Associated with All-Cause Mortality in HIV-Infected Women in the Early and Recent HAART Eras in the Women’s Interagency HIV Study Cohort

**DOI:** 10.1371/journal.pone.0123389

**Published:** 2015-03-26

**Authors:** Matthew R. Gingo, Yingze Zhang, Kidane B. Ghebrehawariat, Jong-Hyeon Jeong, Yanxia Chu, Quanwei Yang, Lorrie Lucht, David B. Hanna, Jason M. Lazar, Mark T. Gladwin, Alison Morris

**Affiliations:** 1 Division of Pulmonary, Allergy, and Critical Care Medicine, Department of Medicine, University of Pittsburgh School of Medicine, Pittsburgh, PA, United States of America; 2 Department of Biostatistics, University of Pittsburgh Graduate School of Public Health, Pittsburgh, PA, United States of America; 3 Department of Epidemiology and Population Health, Albert Einstein College of Medicine, Bronx, NY, United States of America; 4 Department of Medicine, SUNY Downstate Medical Center, Brooklyn, NY, United States of America; 5 Heart, Lung, Blood and Vascular Medicine Institute, University of Pittsburgh and UPMC, Pittsburgh, PA, United States of America; 6 Department of Immunology, University of Pittsburgh School of Medicine, Pittsburgh, PA, United States of America; Fondazione G. Monasterio, ITALY

## Abstract

**Background:**

HIV-infected individuals are at increased risk of right and left heart dysfunction. N-terminal-pro-brain natriuretic peptide (NT-proBNP), a marker of cardiac ventricular strain and systolic dysfunction, may be associated with all-cause mortality in HIV-infected women. The aim of this study was to determine if elevated levels of NT-proBNP is associated with increased mortality in HIV-infected women.

**Design:**

Prospective cohort study.

**Methods and Results:**

We measured NT-proBNP in 936 HIV-infected and 387 age-matched HIV-uninfected women early (10/11/94 to 7/17/97) and 1082 HIV-infected and 448 HIV-uninfected women late (4/1/08 to 10/7/08) in the highly active antiretroviral therapy (HAART) periods in the Women’s Interagency HIV Study. An NT-proBNP >75th percentile was more likely in HIV-infected persons, but only statistically significant in the late period (27% vs. 21%, unadjusted p = 0.03). In HIV-infected participants, NT-proBNP>75th percentile was independently associated with worse 5-year survival in the early HAART period (HR 1.8, 95% CI 1.3–2.4, p<0.001) and remained a predictor of mortality in the late HAART period (HR 2.8, 95% CI 1.4–5.5, p = 0.002) independent of other established risk covariates (age, race/ethnicity, body mass index, smoking, hepatitis C serostatus, hypertension, renal function, and hemoglobin). NT-proBNP level was not associated with mortality in HIV-uninfected women.

**Conclusion:**

NT-proBNP is a novel independent marker of mortality in HIV-infected women both when HAART was first introduced and currently. As NT-proBNP is often associated with both pulmonary hypertension and left ventricular dysfunction, these findings suggest that these conditions may contribute significantly to adverse outcomes in this population, requiring further definition of causes and treatments of elevated NT-proBNP in HIV-infected women.

## Introduction

While highly active antiretroviral therapy (HAART) has reduced mortality related to infectious complications in people with HIV, they are more likely than HIV-uninfected persons to have subclinical cardiovascular disease[1] or be diagnosed with myocardial infarction, congestive heart failure, cardiomyopathy, pulmonary hypertension, and chronic obstructive pulmonary disease (COPD).[2–6] Additionally, HAART may play a role in the development of cardiovascular disease.[7,8] A prior study showed increased cardiac risk factors and rates of acute myocardial infarction in HIV-infected women compared to HIV-uninfected women,[2] suggesting that a better understanding of cardiovascular disease prevalence, risk factors, and outcomes in women with chronic HIV infection is needed.

Several studies have linked markers of inflammation and altered coagulation with increased cardiovascular disease and mortality in HIV.[9–11] C-reactive protein (CRP), interleukin (IL)-6, and D-dimer correlate with traditional markers of HIV infection severity, CD4 count and plasma HIV RNA levels, but are also independently associated with mortality in HIV-infected persons. Similar to HIV-uninfected populations, these markers and other inflammatory markers also correlate with comorbid cardiovascular and pulmonary disease.[12,13]

N-terminal-pro-brain natriuretic peptide (NT-proBNP) is biomarker of cardiac dysfunction that is associated with poor prognosis in several diseases. NT-proBNP is a marker of both left and right heart disease and is released by cardiac myocytes in response to increased wall stress.[14–16] Elevated NT-proBNP levels have been associated with worse outcomes, including mortality, in populations with congestive heart failure (CHF), pulmonary hypertension, sickle cell disease, and COPD.[17–22] Prior work has shown that brain natriuretic peptide is elevated in HIV-infected persons with right and left heart dysfunction.[23–26] Elevated NT-proBNP was also associated with increased risk for cardiovascular events in HIV-infected persons[27]. Additionally, HIV-infected women have been shown to have higher levels of NT-proBNP compared to HIV-uninfected women, but because this has been attributed to an increased prevalence of non-HIV-related factors such as anemia, kidney disease, and hepatitis C infection, NT-proBNP may be a global marker of comorbidity in HIV infection.[28] NT-proBNP has not been evaluated as a marker of mortality in HIV populations. A better understanding of the pathogenesis, risk factors, and outcomes related to cardiac and pulmonary disease in HIV-infected persons is important for both clinical care and research investigations.

We tested the hypothesis that elevated NT-proBNP was associated with mortality in HIV-infected women in the early (1994–1997) and recent (2008) HAART periods using data from the Women’s Interagency HIV Study (WIHS).

## Methods

### Participants

The WIHS cohort has been described previously and is a well-characterized, prospective cohort started in 1994 that is comprised of 3,067 HIV-infected women and 1,070 HIV-uninfected women with high risk for exposure to HIV.[29] We randomly selected half of the HIV-infected participants with plasma samples available from a visit at an early HAART period (10/11/94 to 7/17/97) and half of the HIV-infected participants with a plasma sample available during a late HAART period (4/1/08 to 10/7/08). HIV-uninfected participants with plasma samples available were proportionally matched 1:2 by age at each time period. The institutional review boards of participating institutions (University of Pittsburgh; Women’s Interagency HIV Study Data Management and Analysis Center; Johns Hopkins University; University of California, San Francisco; State University of New York Downstate Medical Center) approved the study protocol, and written informed consent was obtained from all participants.

### Data collection

At six-month visits, age, race/ethnicity, body mass index (BMI), cigarette smoking status, alcohol consumption, use of intravenous drugs in the past 6 months, use of cocaine in the past 6 months, and use of HAART were collected by participant interview. Alcohol consumption was categorized as none, light drinking (<3 drinks/week), moderate drinking (3–13 drinks/week), or heavy drinking (≥14 drinks/week). HAART was defined previously,[30] and antiretroviral medication use was categorized as none, single or combination (not HAART), or current HAART use. HIV status was determined by HIV serological testing at each visit in previously seronegative participants, and participants who were HIV positive at baseline visit were assumed to have duration of HIV infection from time of WIHS enrollment to the date corresponding to NT-proBNP measurement. CD4 count and plasma HIV RNA level were measured at each visit. Deaths were ascertained by National Death Index-Plus match and causes of death were confirmed primarily by AIDS registry data (25%), death certificate review (13%), or both (52%), and defined as previously described.[31]

NT-proBNP was measured in stored plasma using Roche Pro-BNP assay kit with the Elecsys 2010 according to the manufacturer’s instructions (Roche Diagnostics, Indianapolis, IN). When originally collected, blood was drawn into Cell Preparation Tubes (BD Diagnostics, New Jersey, United States) and processed within 6 hours by centrifuging at 1500g at room temperature. Plasma was aliquoted and stored at -80° C in a central repository until assayed in the fall of 2011, whether they were collected in the early (10/11/94 to 7/17/97) or late (4/1/08 to 10/7/08) HAART periods.[32,33] Because of the effect of decreased renal function on NT-proBNP levels, participants with an estimated creatinine clearance below 60 mL/min were excluded from analyses (167 in the early HAART period and 135 in the late HAART period).[34,35]

### Statistical analysis

Covariates were extracted from the visit data corresponding to the time of the plasma sample that was used to measure NT-proBNP was drawn, which we considered the index visit. Data missing from the index visit were imputed from the last observation carried forward if the data were available from a visit within one year prior for the variables creatinine and hemoglobin. If observations had missing data despite these imputations, the observation was not included in the analysis which excluded 204 participants in the early HAART period and 122 from the late HAART period.

Personal characteristics, clinical data, and NT-proBNP levels were described and compared between HIV-infected and HIV-uninfected groups during the early HAART period and late HAART period using t-test, Kruskal-Wallis test, and chi-squared test. NT-proBNP level was dichotomized using the 75^th^ percentile of the cohort to define an elevated level because a normal range or clinical cut-off is not defined for this population. For modeling purposes, continuous covariates were categorized into quartiles.

To determine factors associated with elevated NT-proBNP level in both the early and late HAART periods, multivariable logistic regression was used. In both HAART periods, multivariable logistic regression models were developed for HIV-infected only, HIV-uninfected only, and all (HIV-uninfected and HIV-infected) groups. The following covariates were considered in the analyses: age, race/ethnicity, body mass index, smoking status, alcohol use, illicit drug use, hepatitis C serostatus, hypertension, renal function, hemoglobin, history of AIDS, antiretroviral use, CD4 count, and viral load. In models of HIV-infected women only, the HIV-related variables CD4 count, HIV RNA levels, ART use, and history of AIDS were considered in model creation. Predictors were included in the final multivariable logistic regression models if they met a cutoff p-value of 0.25 in a univariate logistic regression and remained statistically significant at p-value < 0.05 in a multivariable logistic regression that included other significant predictors. A predictor whose exclusion from or inclusion in the final multi-predictor model markedly affected the estimated coefficients of the other predictors was kept in the model regardless of the p-value associated with it.

We used Cox proportional hazards regression to assess association between elevated NT-proBNP level (greater than the 75^th^ percentile) and mortality. Baseline hazards models were substantially different for HIV-infected and HIV-uninfected groups and for the early and late HAART periods, and survival models were developed separately for HIV-infected and HIV-uninfected patient groups in both the early and late HAART periods. The same predictor selection strategy as in the development of the logistic regression models was used to arrive at the final Cox regression models. Time origin was the date of the collection of the plasma sample on which NT-proBNP was measured. Subjects were observed until death, loss to follow-up or latest date of data collection, 7/19/2012. Subjects lost to follow-up were censored at their last visit time. Subjects known to be alive as of the last date of data collection (7/19/2012) were censored at their last visit time.

Due to significant departure from the proportional hazards assumption for the Cox regression model fitted for the entire follow-up period in the HIV-infected group from the early HAART, a Cox regression model was fitted separately for the first 5 years of follow-up period only.[36] Adjusted survival curves under the Cox proportional hazards model were also calculated for each comparison using the average value for covariates in the models. Two sided-values <0.05 were considered significant. To determine the level of NT-proBNP above which is most associated with a decreased survival (greater risk of mortality), we determined a cut-point in the NT-proBNP variable that gives the best separation of the two groups in survival as measured by the log rank statistics using the maxstat package in R (Torsten Hothorn [2014]. maxstat: Maximally Selected Rank Statistics. R package version 0.7–20. http://CRAN.R-project.org/package=maxstat).

To determine if an elevated baseline NT-proBNP was associated with cardiac causes of death, we compared the numbers of death identified as “heart” (myocardial infarction, congestive heart failure, surgery on heart vessels, or other heart disease) or “pulmonary hypertension” vs. all other causes of death in those with elevated NT-proBNP vs. non-elevated NT-proBNP stratified by HIV status and HAART period using the chi-squared test. SAS Version 9.2 (SAS, Inc., Cary, NC) was used for the analysis unless otherwise stated.

## Results

### Participant characteristics

NT-proBNP was measured in 936 HIV-infected and 387 HIV-uninfected individuals in the early HAART period and 1082 HIV-infected and 448 HIV-uninfected individuals in the late HAART periods. HIV-infected participants differed from HIV-uninfected participants in both the early and late HAART periods (Tables 1 and 2).

**Table 1:** Early HAART cohort.

	*HIV-uninfected*	*HIV-infected*	*Overall*	*P-value*
	*(N = 387)*	*(N = 936)*	*(N = 1323)*	
	*Mean (SD)*	*Mean (SD)*	*Mean (SD)*	
	*n (%)*	*n (%)*	*n (%)*	
Age (years)	34.8 (8.3)	36.8 (7.7)	36.2 (7.9)	<0.001
African American	207 (54%)	541 (58%)	748 (57%)	0.15
Hispanic	10 (3%)	39 (4%)	49 (4%)	0.17
Body mass index (kg/m^2^)	28.5 (7.3)	27.0 (6.4)	27.4 (6.7)	<0.001
Smoking Status				0.28
Never smoker	96 (25%)	260 (28%)	356 (27%)	
Current smoker	225 (58%)	499 (53%)	724 (55%)	
Former smoker	66 (17%)	177 (19%)	243 (18%)	
Alcohol use				<0.001
None	184 (48%)	549 (59%)	733 (55%)	
Light	100 (26%)	219 (23%)	319 (24%)	
Moderate	70 (18%)	97 (10%)	167 (13%)	
Heavy	33 (9%)	71 (8%)	104 (8%)	
Illicit drug use, ever	307 (79%)	707 (76%)	1014 (77%)	0.14
Intravenous drug use, ever	57 (15%)	110 (12%)	167 (13%)	0.14
Cocaine use, ever	135 (35%)	270 (29%)	502 (38%)	0.03
Hepatitis C positive	111 (29%)	391 (42%)	502 (38%)	<0.001
Hypertension	91 (24%)	203 (22%)	294 (22%)	0.47
Creatinine (mg/dL)	0.87 (.16)	0.88 (.17)	0.88 (.17)	0.53
Hemoglobin (gm/dL)	13.1 (1.2)	12.3 (1.5)	12.5 (1.5)	<0.001
History of AIDS		607 (65%)		n/a
Antiretroviral medication use				n/a
None		414 (44%)		
Prior HAART		483 (52%)		
Current HAART		39 (4%)		
HAART duration		0.00 (.05)		n/a
CD4 counts: Current, at baseline pulmonary	1086 (388)	356 (281)	565 (457)	<0.001
CD4 counts: Mean over follow-up period	1256 (412)	439 (321)	673 (508)	<0.001
CD4 counts: Nadir over follow-up period	910 (321)	289 (235)	467 (384)	<0.001
Plasma HIV RNA level (copies/mL)		18x10^3^ (1x10^5^)		n/a
HIV RNA detectable		842 (90%)		n/a
NT-proBNP (above 3^rd^ Quartile)	86 (22%)	245 (26%)	331 (25%)	0.13

**Table 2:** Late HAART cohort.

	*HIV-uninfected*	*HIV-infected*	*Overall*	*P-value*
	*(N = 448)*	*(N = 1082)*	*(N = 1530)*	
	*Mean (SD)*	*Mean (SD)*	*Mean (SD)*	
	*n (%)*	*n (%)*	*n (%)*	
Age (years)	40.9 (9.7)	44.5 (8.2)	43.4 (8.8)	<0.001
African American	287 (64%)	614 (57%)	901 (59%)	0.008
Hispanic	61 (14%)	156 (14%)	217 (14%)	0.68
Body mass index (kg/m^2^)	31.5 (8.7)	29.6 (7.5)	30.2 (7.9)	<0.001
Smoking Status				<0.001
Never smoker	112 (25%)	360 (33%)	472 (31%)	
Current smoker	220 (49%)	390 (36%)	610 (40%)	
Former smoker	116 (26%)	332 (31%)	448 (29%)	
Alcohol use				<0.001
None	193 (43%)	682 (63%)	875 (57%)	
Light	156 (35%)	276 (26%)	432 (28%)	
Moderate	74 (17%)	92 (9%)	166 (11%)	
Heavy	25 (6%)	32 (3%)	57 (4%)	
Illicit drug use, ever	295 (66%)	516 (48%)	811 (53%)	<0.001
Intravenous drug use, ever	40 (9%)	71 (7%)	111 (7%)	0.10
Cocaine use, ever	171 (38%)	293 (27%)	464 (30%)	<0.001
Hepatitis C positive	73 (16%)	265 (25%)	338 (22%)	<0.001
Hypertension	135 (30%)	341 (32%)	476 (31%)	0.59
Creatinine (mg/dL)	0.80 (.15)	0.79 (.16)	0.79 (.16)	0.29
Hemoglobin (gm/dL)	12.6 (1.3)	12.4 (1.4)	12.5 (1.4)	0.02
History of AIDS		478 (44.2%)		n/a
Antiretroviral medication use				n/a
None		221 (20%)		
Prior HAART		12 (1%)		
Current HAART		849 (79%)		
HAART duration		8.04 (3.8)		n/a
CD4 counts: Current, at baseline pulmonary	1081 (394)	520 (290)	684 (413)	<0.001
CD4 counts: Mean over follow-up period	1443 (500)	821 (387)	1003 (509)	<0.001
CD4 counts: Nadir over follow-up period	754 (274)	224 (165)	379 (315)	<0.001
Plasma HIV RNA level (copies/mL)*		80.0 (1x10^3^)		n/a
HIV RNA detectable		426 (39%)		n/a
NT-proBNP (above 3^rd^ Quartile)	95 (21%)	288 (27%)	383 (25%)	0.03

### Predictors of elevated NT-proBNP

In the entire cohort, the 75^th^ percentile of NT-proBNP level in the early HAART period was 58.7ng/L. An elevated (>75^th^ percentile) NT-proBNP level was associated with advanced age, African American race, lower body mass index, smoking, Hepatitis C seropositive status, lower hemoglobin level, and presence of hypertension (Table 3—early HAART period). In the entire cohort during the late HAART period, the 75^th^ percentile of NT-proBNP level was 60.6ng/L, and an elevated NT-proBNP level was associated with same findings as the early period except African American race and hypertension (Table 3 —late HAART period). The presence of elevated NT-proBNP levels was higher among HIV-infected women than in uninfected women in both the early and late HAART periods, but this difference was only statistically significant in the later period (27% of HIV-infected participants with elevated NT-proBNP vs. 21% of HIV-uninfected, unadjusted p = 0.03). HIV status did not remain associated with elevated NT-proBNP level in the late HAART period after adjustment in the multivariable model. In HIV-infected participants, during the early and late HAART periods, the 75^th^ percentile NT-proBNP levels were 60.8ng/L and 63.3ng/L, respectively. Factors associated with elevated NT-proBNP (>75^th^ percentile) in HIV-infected participants were similar in the early and late HAART periods except that African-American race was significantly associated with elevated NT-proBNP level in the early period, and in the late period, light drinking was associated with lower and heavy drinking associated with higher NT-proBNP levels (Table 4). No HIV-related factors were found to be associated with NT-proBNP level, and cocaine use was not associated with elevated NT-proBNP.

**Table 3:** Factors associated with an elevated BNP (>75^th^ percentile) in the entire cohort in early and late HAART periods.

**Early HAART period**	**OR**	**95% CI**	**p-value**
HIV infection	0.84	03.62–1.15	0.27
Age Q2 (ref = Q1)	2.08	1.39–3.16	<0.001
Age Q3	2.27	1.51–3.45	<0.001
Age Q4	3.06	2.04–4.65	<0.001
African American (ref = white, non-Hispanic)	0.47	0.33–0.66	<0.001
Hispanic (ref = white, non-Hispanic)	0.61	0.41–0.91	0.01
Hemoglobin Q2 (ref = Q1)	0.44	0.31–0.63	<0.001
Hemoglobin Q3	0.32	0.21–0.47	<0.001
Hemoglobin Q4	0.25	0.17–0.36	<0.001
Hypertension	1.61	1.17–2.19	0.003
**Late HAART period**	**OR**	**95% CI**	**p-value**
HIV infection	1.13	0.85–1.51	0.41
Age Q2 (ref = Q1)	1.72	1.17–2.55	0.006
Age Q3	1.89	1.28–2.82	0.002
Age Q4	2.39	1.59–3.63	<0.001
Hypertension	1.72	1.30–2.28	<0.001
BMI Q2 (ref = Q1)	0.66	0.47–0.93	0.02
BMI Q3	0.49	0.34–0.69	<0.001
BMI Q4	0.69	0.49–0.97	0.03
Hepatitis C antibody positive	1.52	1.13–2.05	0.006
Hemoglobin Q2 (ref = Q1)	0.72	0.51–1.01	0.06
Hemoglobin Q3	0.49	0.35–0.70	<0.001
Hemoglobin Q4	0.43	0.30–0.62	<0.001
African American (ref = white, non-Hispanic)	0.66	0.48–0.91	0.01
Hispanic (ref = white, non-Hispanic)	0.82	0.56–1.19	0.3
Smoking—current (ref = never smoking)	1.37	1.00–1.89	0.050
Smoking—former	0.86	0.61–1.20	0.36

Q = quartile; BMI = body mass index.

**Table 4:** Factors associated with an elevated NT-proBNP (>75^th^ percentile) in HIV-infected participants in the early and late HAART periods.

**Early HAART period**	**OR**	**95% CI**	**p-value**
Hypertension	1.89	1.31–2.74	<0.001
Hepatitis C antibody positive	2.14	1.56–2.96	<0.001
Hemoglobin Q2 (ref = Q1)	0.40	0.26–0.60	<0.001
Hemoglobin Q3	0.26	0.17–0.41	<0.001
Hemoglobin Q4	0.16	0.10–0.26	<0.001
African American (ref = white non-Hispanic)	0.60	0.39–0.89	0.02
Hispanic (ref = white non-Hispanic)	0.78	0.46–1.21	0.31
**Late HAART period**	**OR**	**95% CI**	**p-value**
Hypertension	1.77	1.31–2.38	<0.001
Drinking, light (ref = abstinent)	0.66	0.45–0.93	0.03
Drinking, moderate	1.30	0.77–2.10	0.31
Drinking, heavy	2.81	1.25–5.73	0.008
Hepatitis C antibody positive	2.25	1.58–2.99	<0.001
Hemoglobin Q2 (ref = Q1)	0.87	0.58–1.28	0.48
Hemoglobin Q3	0.52	0.35–0.79	0.002
Hemoglobin Q4	0.49	0.32–0.73	<0.001

Q = quartile.

### Association of NT-proBNP level with mortality

**Fig 1:**
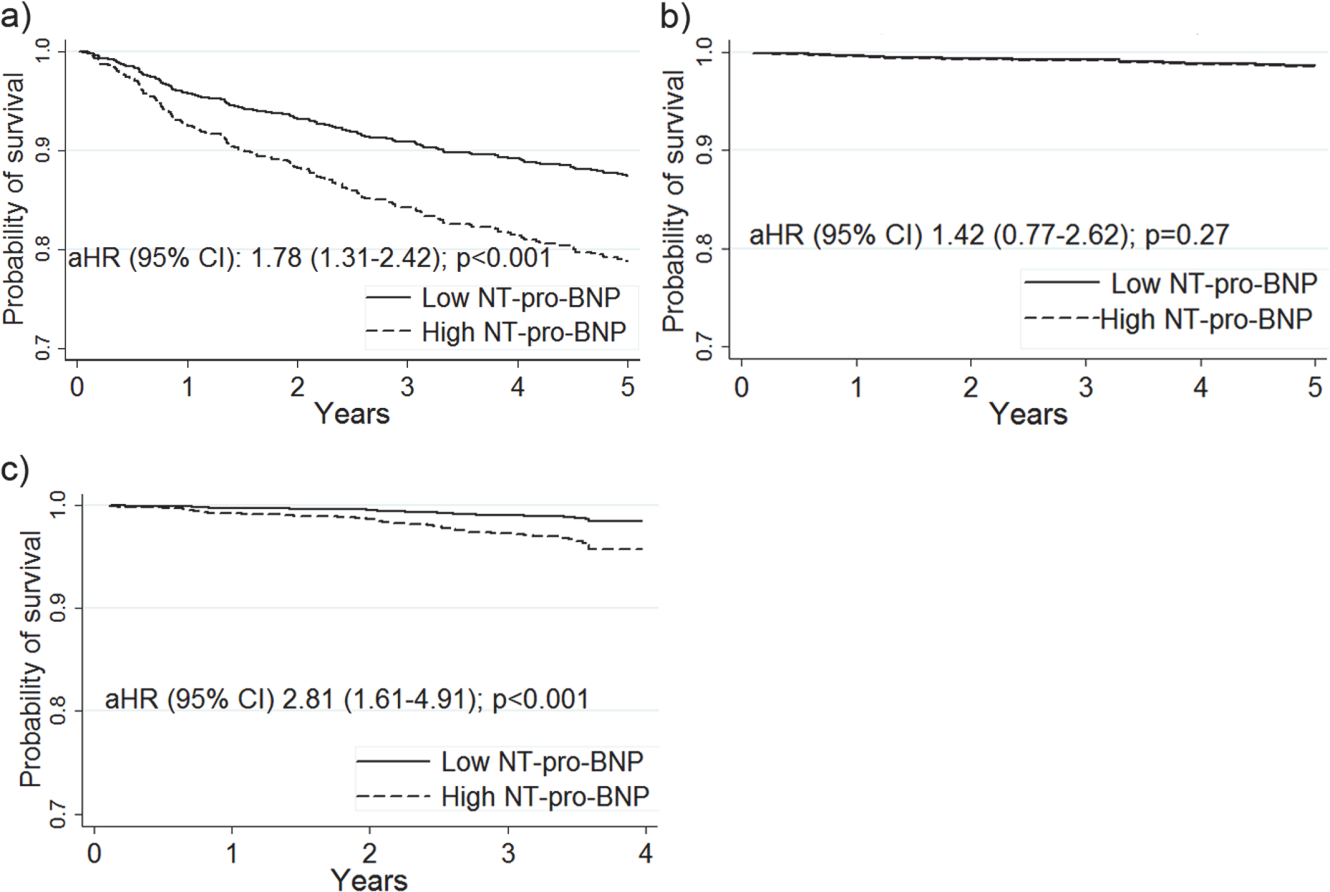
a) Cox adjusted survival curves for HIV-infected participants with NT-proBNP≤75th percentile (solid) and HIV-infected with NT-proBNP>75th percentile (dash) during the early HAART period. b) Cox adjusted survival curves for HIV-uninfected participants with NT-proBNP≤75^th^ percentile (solid) and HIV-uninfected participants with NT-proBNP>75^th^ percentile (dash) during the early HAART period. c) Cox adjusted survival curves for HIV-infected participants with NT-proBNP≤75^th^ percentile (solid) and HIV-infected participants with NT-proBNP>75^th^ percentile (dash) during the late HAART period. aHR = adjusted hazard ratio; 95% CI = 95% confidence interval for the adjusted hazard ratio.

We analyzed mortality in relation to NT-proBNP levels in HIV-infected participants in the early (n = 936; 403 mortality events) and late (n = 1082; 55 mortality events) HAART periods and in the HIV-uninfected participants in the early HAART period (n = 387; 48 mortality events). There were too few deaths in the HIV-uninfected group to assess an association with NT-proBNP level in the late HAART period (n = 4). In HIV-infected participants, NT-proBNP>75^th^ percentile was independently associated with greater 5-year mortality in the early (HR 1.78, 95% CI 1.31–2.42, p<0.001) and in the late (HR 2.81, 95% CI 1.61–4.91, p = 0.002) periods (**Fig. 1**). These associations were independent of other factors associated with mortality including HIV-related variables such as HAART use, CD4 T-lymphocyte cell count, and HIV RNA levels. In HIV-uninfected women in the early period, elevated NT-proBNP level was not associated with mortality. In the early period (from 10/11/94 to 7/17/97) the optimal cut-point above which was associated with worse survival was 70.2 ng/L which was the 80th percentile. In the late period (from 4/1/08 to 10/7/08) the optimal cut-point was 114.8 ng/L which corresponded to the 90th percentile.

### Mortality outcomes

There were very few deaths clinically attributed to cardiac disease in the early and late HAART periods in either the HIV-infected or HIV-uninfected participants. In the HIV-infected participants from the early HAART period, 3% in the low NT-proBNP group and 5% in the elevated NT-proBNP group had causes of death related to defined cardiac disease (p = 0.43). In the HIV-uninfected participants from the early HAART group, 3% in the low NT-proBNP and 12% from the high NT-proBNP group had cardiac related deaths (p = 0.28). In the HIV-infected participants from the late HAART period, there have been zero deaths attributed to cardiac causes in the low NT-proBNP group, but 9% in the elevated NT-proBNP group had causes of death related to cardiac disease (p = 0.24).

## Discussion

This analysis is the first to identify NT-proBNP, a marker of cardiac stress, as independently associated with mortality in HIV-infected women. Elevated NT-proBNP was not related to HIV severity such as CD4 count or plasma HIV RNA level, and the association of NT-proBNP with mortality was independent of CD4 count and plasma HIV RNA level. Additionally, the effect size of elevated NT-proBNP on mortality in the HIV-infected group was greater in the late period where HAART use was more common. Because NT-proBNP is a sensitive marker for cardiac wall stress (which may be left- or right-sided), these findings could reflect cardiac or pulmonary vascular disease as a significant contributor to HIV-related comorbidity and mortality in the HIV population, but recent studies illustrating that the production/secretion of natriuretic peptide is also strongly influenced by other inflammatory, physiologic, and toxic processes may also help explain our findings in the HIV population.

As in the general population, elevated NT-proBNP levels reflect cardiac and pulmonary vascular disease in HIV-infected individuals. Several studies in HIV-infected populations have shown elevated BNP associated with increased rates of heart failure, cardiomyopathy, coronary artery disease, pulmonary hypertension, and left and right heart abnormalities measured by echocardiogram and magnetic resonance imaging.[24,25,27,37] A prior publication in a smaller subgroup from the WIHS cohort found HIV-infected participants had higher NT-proBNP levels, and NT-proBNP was associated with typical risk factors for cardiac and pulmonary disease such as age, race, body mass index, smoking, illicit drug use, hepatitis C infection, hemoglobin, and hypertension.[28] NT-proBNP may also be produced and secreted in response to mechanisms related to other common phenomenon in the HIV population such as increased inflammatory cytokines important in the pathogenesis of cardiomyopathy—interleukin-1, interleukin-6, and tumor necrosis factor-α,[38,39] exposure to toxins such as cocaine which has cardio-toxic effects,[40,41] and lipotoxicity which can lead to metabolic and cardiac disease.[42,43] Adjusting for other variables, no HIV-related variables were associated with elevated NT-proBNP, and HIV infection was not an independent predictor for elevated NT-proBNP after adjusting for anemia, hepatitis C infection, and impaired kidney function, which were more common in the HIV-infected participants. Our findings of associations of NT-proBNP in HIV-infected women were similar to prior work, but this is the first study, to our knowledge, to assess the association between NT-proBNP and mortality in HIV.

We demonstrated that an elevated NT-proBNP was associated with increased all-cause mortality in HIV-infected participants, but was not significantly associated with mortality in the HIV-uninfected group from the same cohort and similar HIV risk factors. Elevated BNP levels are associated with increased mortality in patients with cardiac and pulmonary diseases,[17–20,22] and NT-proBNP was also a marker of mortality and cardiovascular events in a community sample, the Framingham Offspring Study.[44] The participants in our study were much younger than the Framingham Offspring study (35 years old in the early HAART period and 43 in the late HAART period compared to 60 years old in Framingham) suggesting that HIV-infected individuals may have an accelerated risk of NT-proBNP-related pathology. The younger age may also explain why we did not see a significant association between elevated NT-proBNP and mortality in the HIV-uninfected participants.

NT-proBNP was not associated with degree of immunosuppression and its relationship with mortality was seen in both the early and late HAART periods. Baseline CD4 count and plasma HIV RNA level were not associated with NT-proBNP in either the early HAART period, when few participants were on effective HAART or virally-suppressed, or in the late HAART period, when most were on successful HAART. These findings contrast with prior studies of biomarkers associated with mortality in HIV.[9,12] For example, biomarkers of inflammation and coagulation such as CRP, IL-6, and D-dimer were often associated with greater viral levels or lower CD4 counts;[9,10,12,45] however, NT-proBNP, a protein derived from cardiac myocytes, was not associated with these traditional HIV markers. Lack of association with traditional HIV markers may indicate that a mechanism independent of severity of HIV disease is involved in the association between elevated NT-proBNP levels and mortality.

The underlying cause of the relationship between mortality in HIV infection and elevated NT-proBNP is unclear. Several studies in HIV have shown elevated BNP is associated with cardiac dysfunction,[24,25,37] and because inflammation, cocaine use, and metabolic disease can be other mechanisms leading to increased NT-proBNP and are common in HIV-infected persons, NT-proBNP may be a surrogate risk marker of ongoing cardiac damage from chronic inflammation, illicit drug use, or metabolic disease in the HIV-population.[38–43] We assessed the cause of death in relationship to elevated NT-proBNP level to see if the association was related to cardiac disease specifically, but very few causes of death were clinically attributed to cardiac cause in the early and late HAART periods. This study and others have also found that hepatitis C infection and anemia are independently associated with elevated NT-proBNP, and renal disease is known to affect NT-proBNP levels.[34,46,47] We excluded persons with reduced renal function and used the calculated creatinine clearance in modeling to eliminate potential confounding from significant renal disease that would alter NT-proBNP level and mortality; however, there may be residual confounding from these factors that could explain the association we find between NT-proBNP level and mortality. Further study in a prospective manner may be necessary to determine exactly how NT-proBNP links HIV infection and mortality. As NT-proBNP is significantly associated with mortality risk and is a readily available and inexpensive test, it may be a useful marker to determine elevated risk of death in the HIV-infected population. Further evaluation of NT-proBNP as a screening test for cardiopulmonary disease in the HIV-infected population is warranted.[28]

Our study has several unique strengths as well as important limitations. We expand on prior findings in the WIHS cohort that NT-proBNP is associated with co-morbidities and show there is also a significant association with mortality. This study benefits from the strength of a well-characterized and prospectively followed cohort of HIV-infected and high-risk HIV-uninfected women with similar baseline characteristics. Also, we were able to assess the effect treatment has on the relationship between NT-proBNP and mortality by including both an early HAART period where viral suppression was not common and a later HAART period with high prevalence of antiretroviral use and viral suppression. This cohort is unique in that it is strictly women and comprised primarily of minorities with a history of engaging in high-risk behavior, predominantly illicit drug use. It is unknown if the current findings would be applicable to men (however, since men are at risk for earlier cardiovascular disease, it may be more applicable) or those without illicit drug use or intravenous drug use behaviors. We were also not able to look at specific diseases that may contribute to the NT-proBNP such as pulmonary hypertension, atherosclerosis, cardiomyopathy, etc. Another limitation is that NT-proBNP was not measured at the time the plasma sample was obtained. Degradation of the NT-proBNP peptide in storage is possible and could influence the absolute level of NT-proBNP. Storage in -80° C should limit degradation, and our early period levels were expected to be lower due to younger age of the cohort at that time, and NT-proBNP correlated with other biomarkers as expected (hemoglobin, creatinine, etc). In addition, the relative relationships should be expected to be unaltered by the degradation.

In conclusion, we have found a novel biomarker for mortality in HIV-infected women that is independent of CD4 counts or plasma HIV RNA levels. As NT-proBNP is a marker of cardiovascular and pulmonary disease in the general population and this HIV cohort is relatively younger than those in general population-based studies, our findings suggest that HIV-infected women may be at increased risk for cardiopulmonary comorbidities that could lead to increases in mortality. Elevated NT-proBNP in HIV-infected women may be a useful marker for risk stratification and aid in the investigation of comorbid complications of HIV.
